# Koala retrovirus load and non-A subtypes are associated with secondary disease among wild northern koalas

**DOI:** 10.1371/journal.ppat.1010513

**Published:** 2022-05-19

**Authors:** Michaela D. J. Blyton, Michael Pyne, Paul Young, Keith Chappell

**Affiliations:** 1 The University of Queensland, School of Chemistry and Molecular Biosciences, St Lucia, Queensland, Australia; 2 Currumbin Wildlife Hospital and Foundation, Currumbin, Queensland, Australia; 3 The University of Queensland, Australian Institute of Bioengineering and Nanotechnology, St Lucia, Queensland, Australia; University of Illinois at Chicago College of Medicine, UNITED STATES

## Abstract

Koala Retrovirus (KoRV) has been associated with neoplasia in the vulnerable koala (*Phascolarctos cinereus*). However, there are conflicting findings regarding its association with secondary disease. We undertook a large-scale assessment of how the different KoRV subtypes and viral load are associated with *Chlamydia pecorum* infection and a range of disease pathologies in 151 wild koalas admitted for care to Currumbin Wildlife Hospital, Australia. Viral load (KoRV *pol* copies per ml of plasma) was the best predictor of more disease pathologies than any other KoRV variable. The predicted probability of a koala having disease symptoms increased from 25% to over 85% across the observed range of KoRV load, while the predicted probability of *C*. *pecorum* infection increased from 40% to over 80%. We found a negative correlation between the proportion of *env* deep sequencing reads that were endogenous KoRV-A and total KoRV load. This is consistent with suppression of endogenous KoRV-A, while the exogenous KoRV subtypes obtain high infection levels. Additionally, we reveal evidence that the exogenous subtypes are directly associated with secondary disease, with the proportion of reads that were the endogenous KoRV-A sequence a negative predictor of overall disease probability after the effect of KoRV load was accounted for. Further, koalas that were positive for KoRV-D or KoRV-D/F were more likely to have urogenital *C*. *pecorum* infection or low body condition score, respectively, irrespective of KoRV load. By contrast, our findings do not support previous findings that KoRV-B in particular is associated with *Chlamydial* disease. Based on these findings we suggest that koala research and conservation programs should target understanding what drives individual differences in KoRV load and limiting exogenous subtype diversity within populations, rather than seeking to eliminate any particular subtype.

## Author summary

With the koala now listed as vulnerable in Australia and local population declines of greater than 80% in part due to disease, it is now critically important to establish the role koala retrovirus (KoRV) plays in disease to guide future conservation efforts. While KoRV has previously been linked to increased rates of cancer in koalas, it is less clear whether it also increases the koalas’ susceptibility to other infections. We assessed how KoRV is associated with *Chlamydia* and a range of other diseases. We found that the amount of KoRV circulating in the blood was strongly associated with disease. KoRV exists as both a heritable virus in the koala’s genome and as an infectious virus. We found that the infectious forms of the virus are associated with higher levels of KoRV in the blood and directly with disease. These findings highlight the major role KoRV is likely to play in the high rate of disease in northern koala populations, which is contributing to their decline. We suggest that koala research and conservation should target understanding what drives differences among koalas in the amount of KoRV circulating in the blood and on limiting the spread of different KoRV subtypes between populations.

## Introduction

Retroviruses are known to cause severe disease through integration of their genetic material into the host’s genome, which can lead to carcinogenesis [1]; and through the infection and damage of somatic cells. Retroviruses commonly infect the rapidly dividing cells of the immune system, causing immune suppression and predisposing the host to secondary diseases [2–4]. Koala Retrovirus (KoRV) is one such gammaretrovirus that has been associated with both neoplasia and secondary disease in koalas (*Phascolarctos cinereus*) [5–8]. However, the complexity of KoRV’s biogeography, transmission dynamics and subtype diversity has led to apparently conflicting conclusions regarding KoRV’s disease association. With the koala now listed as vulnerable in Australia and local population declines of greater than 80% due to habitat loss, wildfire and disease [9,10], it is now critically important to establish the role KoRV plays in disease to guide future treatment, rehabilitation and population management efforts. In particular, establishing how KoRV influences the prevalence and severity of secondary diseases such as *Chlamydia* is highly pertinent to the conservation of wild populations. Infection with *Chlamydia percorum* is the leading cause of conjunctivitis and urogenital pathologies in koalas and is a key driver of population decline in northern Australian koalas [10]. In this study we undertook a large-scale assessment of how the different KoRV subtypes and viral load are associated with secondary disease in wild koalas.

Unlike the majority of retroviruses, KoRV exists as both an endogenous and exogenous virus [11–15]. The intact endogenous form of the virus (that is integrated into the koalas’ germline and inherited) is found in all koalas in the northern Australian states of Queensland and New South Wales but is absent from the southern states of Victoria and South Australia [8,16–19]. Only KoRV subtype A is known to be endogenous [11,20]. The activity of such endogenous retroviruses is generally supressed by host genetic mechanisms including transcriptional silencing and post-transcriptional RNA degradation guided by piRNAs. In the koala, KoRV specific piRNAs have been detected in germline cells and are proposed to transcriptionally silence endogenous KoRV-A integrations [21]. However, transcriptional silencing of germline integrations is incomplete and re-integration of KoRV into somatic cells within certain tissues is frequent [21,22]. Additionally, KoRV-A is present in southern koalas at low copy numbers per host cell, which is suggestive of exogenous infection [16,17], and appears to be exogenously transmitted from mothers’ to their joeys in northern koalas [23]. The ability of KoRV-A to cause disease is difficult to assess in northern populations as it is ubiquitous. Nonetheless, KoRV-A insertion sites have been linked to increased cancer rates [1] and KoRV-A positivity has been associated with urogenital infection and poor body condition in Victorian koalas [8].

Classification of KoRV subtypes is based on the amino acid sequence for the receptor binding domain of the envelope protein (coded for by the *env* gene) that mediates viral entry into the host cell [24]. In addition to KoRV-A, 12 other subtypes (B-M) have been described that are thought to be exogenously transmitted and have not been detected as endogenous proviral insertions [19,20,23,24]. KoRV subtype diversity was first thought to be limited, with subtypes B-F first isolated from captive koalas [5,25,26] and only KoRV-B has been routinely screened for by PCR in wild and captive populations [6,7]. However, next generation sequencing of the *env* gene has revealed extensive subtype diversity [18,19,23,24,27–30]. Blyton et al. [19] has shown that with the exception of KoRV-D, these subtypes are locally prevalent but geographically restricted, suggesting that further yet to be sampled diversity exists. There is some evidence that KoRV-B (also previously referred to as J) may be more virulent than KoRV-A as it has been associated with Chlamydiosis and neoplasia in southern Queensland koalas [5–7]. However, it is not clear if this association is due to the exogenous nature of KoRV-B or a particular genetic attribute of the subtype. Little is known about the disease association of the other exogenous subtypes. One study in captive koalas found that the non-KoRV-A subtypes may be associated with increased cancer rates [31]. By contrast, KoRV-D expression levels have been found to be higher in healthy koalas compared to those that developed *Chlamydial* disease [32]. Although, the small sample size of that study and the extensive genetic diversity within KoRV-D calls into question the generality of that finding. Taken together, it is clear that further evaluation of how the different subtypes relate to secondary disease is urgently needed.

Irrespective of KoRV subtype, higher levels of KoRV viremia are expected to be associated with disease. Indeed, in three koala cohorts studied [31,33–35] an association between plasma viral load and neoplasia has been found. However, in the case of secondary diseases, previous studies have produced mixed findings. In a small number of captive koalas in Japan, KoRV RNA was only detectable in the blood of a sick koala but not healthy koalas [36]. Further a study in Victorian koalas [8] found higher KoRV loads in females with urogenital tract disease. In a population of koalas in South Australia, plasma viral load was associated with the severity of chlamydial disease, however, the same association was not seen in Queensland koalas in that study [34,35]. Further, no association between either proviral or plasma viral loads and urogenital or ocular *Chlamydia percorum* loads or disease were seen in that study [35]. A small scale study of wild south-east Queensland koalas also found no association between viral load and Chlamydial disease [7] and another found higher expression of KoRV, primarily attributed to KoRV-D, in healthy koalas than those with Chlamydiosis [28]. Therefore, to adequately assess how KoRV plasma load is related to secondary disease in particular, comprehensive studies with large sample sizes are needed.

Therefore, to comprehensively investigate how plasma viral load and the different KoRV subtypes are associated with secondary disease we collected blood samples from 151 wild koalas admitted to Currumbin Wildlife Hospital (CWH), Australia, for treatment of disease or injury. Veterinary records kept by CWH were used to score the sampled koalas for the presence and extent of a set of gross disease pathologies (S1 Table). The presence of urogenital and ocular *Chlamydia pecorum* infection was also determined by CWH using loop-mediated isothermal amplification [37]. We determined KoRV plasma viral loads (viremia) and subtype profiles for the sampled koalas by qPCR and *env* deep sequencing, respectively, using cDNA synthesised from total extracted plasma RNA. The large sample size and in-depth analysis of these samples allowed us to 1) determine how KoRV load is associated with the exogenous subtypes; and 2) to disentangle if KoRV load, particular exogenous subtypes or both are associated with secondary disease. We show clear evidence that both KoRV load and the exogenous subtypes are strongly associated with secondary disease, providing direction for the conservation management of the koala.

## Results

### KoRV subtype profiles

#### Env sequence diversity

Across the 151 sampled koalas, 10,267 unique sequence clusters were identified for a 500 bp region of the KoRV *env* gene containing the previously identified hypervariable region (HVR) [24], amplified from vRNA/cDNA extracted from cellular free plasma (after *de novo* clustering of the quality controlled deep sequencing reads at 97% similarity and removal of singletons). This level of sequence diversity is an order of magnitude higher than found for proviral (DNA) sequences from a comparable number of koalas distributed across eastern Australia [19]. This suggests that KoRV undergoes high levels of transcriptional mutation and recombination to generate high levels of vRNA diversity. Sarker et al [30], also observed a trend for higher sequence diversity in vRNA sequences when compared to provirus, although the difference was not as pronounced as in this study and was not significant.

Almost half of these sequences (47.3%) were classified as non-functional including 3,467 that contained nonsense mutations, 1,051 that contained deletions, 228 that were truncated and 110 containing frameshift mutations. Intact KoRV-A sequences accounted for 634 (6.2%) of clusters, while, there were 711 (6.9%) intact KoRV-B sequence clusters and 1,928 (18.8%) KoRV-D sequence clusters. A small number of KoRV-H (n = 104) sequence clusters were also identified, while none of the other published subtypes (C, E-G, I, K, L or M) were detected.

Intermediate sequences between several different subtypes were also detected from the *in silico* translated amino acid sequences, where the first section of the hypervariable region showed homology to one subtype and the remaining section resembled another subtype. These intermediate sequences included 14 A/D intermediates, two A/F intermediates and one B/D intermediate (S1 Fig). With the exception of the A/F intermediates, these intermediate sequences were only found in koalas that also carried the two parent subtypes, suggesting they were the result of within koala recombination. There were also a large number (n = 1,844; 18.0%) of sequence clusters that were identified as D/F intermediate sequences. The first section of these sequences were consistent with KoRV-D, while, the end of the hypervariable region showed homology to KoRV-F. Unlike the other intermediate sequences, the abundance of D/F intermediate sequences and the absence of KoRV-F from the sampled koalas suggests that their immediate origin is unlikely to be within koala recombination.

The remaining 169 sequence clusters had low homology to the previously published subtypes A-M (maximum amino acid identity of HVR to subtype reference sequences = 75.3). Among these clusters four groups of similar sequences (average HVR amino acid identity within groups = 87.5, 84.9, 83.9 and 81.2 respectively) could be identified that may represent new KoRV subtypes, containing 35, 16, 23 and 10 sequence clusters respectively (S2 Fig). However, we have not confirmed that these sequences are present as proviral insertions and therefore designate them as groups 1–4 for the purposes of this study. The remaining 85 sequence clusters were distinct from the other sequence groups and each other and were classified as undefined subtypes.

#### Subtype prevalence and relative abundance

**Fig 1:**
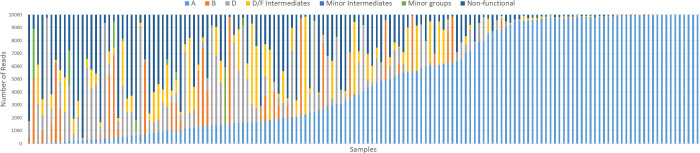
Number of *env* reads assigned to each KoRV group for each sample.

KoRV-A sequence clusters were detected in all sampled koalas, however, there was extensive variation among individuals in their relative abundance, with the percentage of reads attributed to KoRV-A ranging from 0.38–99.98% (Fig 1). In an exploratory analysis to explain this variation, we found that koalas carrying a greater number of subtypes had a lower relative abundance of KoRV-A (R^2^ = 0.21; p < 0.001). All koalas expressed KoRV-A sequences that clustered with the original KoRV-A endogenous sequence [11,38] and in general, these accounted for the vast majority of KoRV-A reads detected within a koala (median = 98.1%; interquatile range = 89.2–99.7%; S3 Fig). However, all koalas also expressed additional KoRV-A sequence variants that accounted for a small proportion of the total reads (maximum = 5.1%, excluding two outliers of 60.4% and 80.4%). Non-functional sequence clusters were also found in all sampled koalas, with vast differences among koalas in their relative abundance, ranging from 0.01% to 95.5% (median = 11.2%; interquartile range = 1.5–38.6%; Fig 1).

KoRV-D was the most prevalent non-KoRV-A subtype, closely followed by KoRV-D/F, with both variants found in the vast majority of koalas (Table 1). KoRV-B was also detected in over half the sampled koalas (Table 1). All three of these subtypes reached high relative abundances in some koalas, accounting for over 75% of reads in some instances (Fig 1 and Table 1). KoRV-H and the four new HVR variants were only detected in a small proportion of koalas (≤6%) and the undefined sequences only reached a maximum combined relative abundance of 7.45% within koalas (Table 1). Therefore, these groups were pooled into a ‘minor groups’ category for the disease association analysis. With the exception of the D/F intermediates, intermediate sequences accounted for <1% of reads within koalas (Table 1) and were therefore not included in further analysis.

**Table 1:** Prevalence and relative abundance of KoRV subtypes.

Subtype/sequence group	Number of koalas (%)	Maximum percentage of reads within a koala
A	151 (100)	99.98
B	83 (55.0)	83.69
D	138 (91.4)	96.6
H	9 (6.0)	43.23
A/D Intermediates	44 (29.1)	0.13
A/F Intermediates	2 (1.3)	0.03
B/D Intermediates	3 (2.0)	0.03
D/F Intermediates	132 (87.4)	76.08
Group 1	2 (1.3)	6.50
Group 2	5 (3.3)	20.31
Group 3	2 (1.3)	38.21
Group 4	1 (0.7)	0.63
Undefined	54 (35.8)	7.45
Non-functional	151 (100)	95.54

### Association between plasma viral loads and subtype diversity

KoRV *pol* plasma loads varied by 4 orders of magnitude among koalas, ranging from 4.15 to 8.05 log10 copies per ml (as estimated by qPCR of a fragment of the KoRV *pol* gene [36]; mean ± SD = 5.71 ± 0.91). There was a weak but significant tendency for KoRV load to increase with koala age as estimated from tooth wear (as determined by linear regression, R^2^ = 0.059, coefficient = 0.087, t = 2.14, p = 0.036; S4 Fig). However, the predicted increase in KoRV load with age (<30% increase per year), is far below the variation seen within similarly aged koalas (typically 2–3 orders of magnitude). There was no significant difference in KoRV load between the sexes (Unpaired t-test; t = -0.57, p = 0.568).

**Fig 2:**
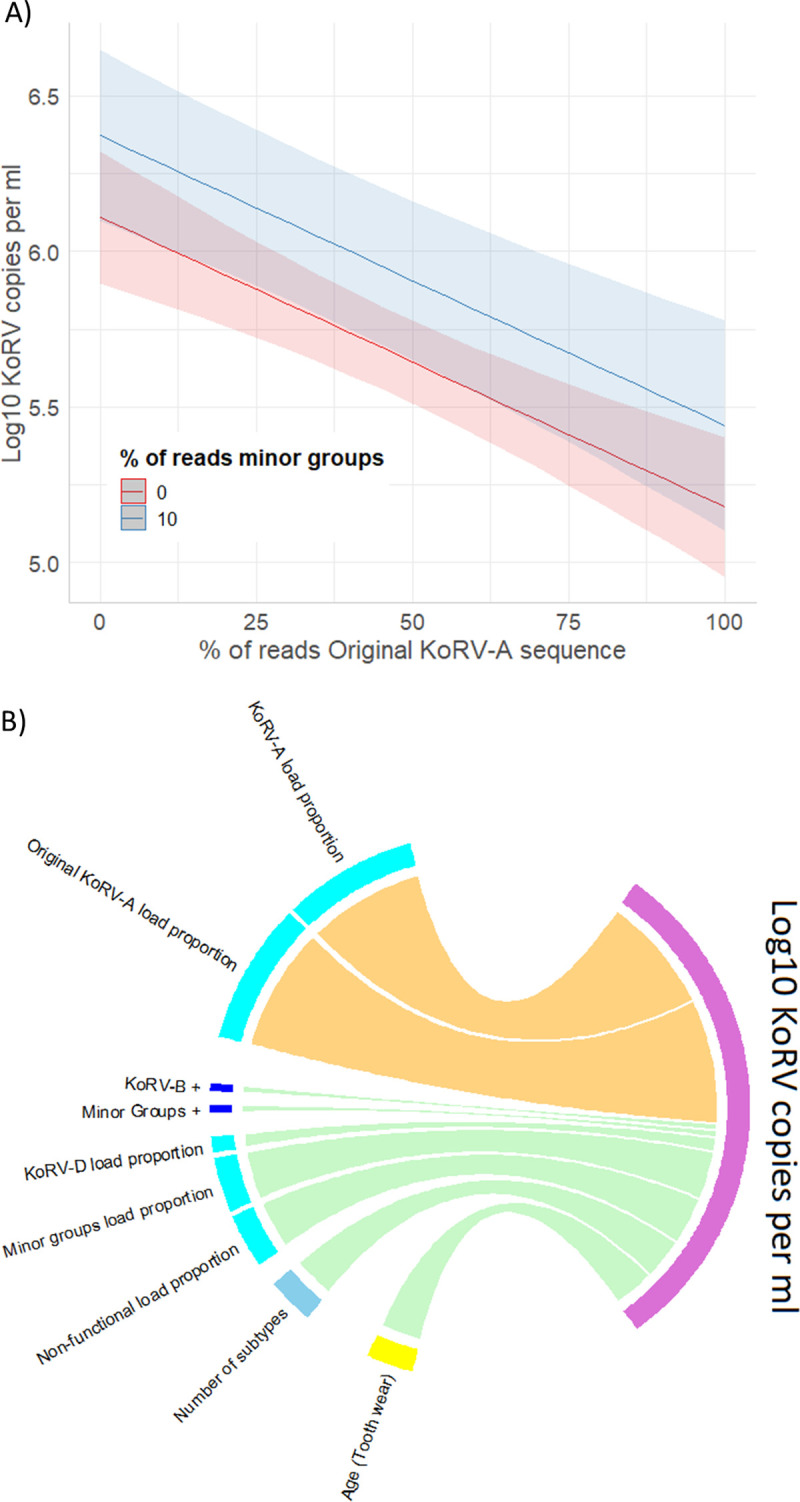
A) The best model of log10 KoRV *pol* copies per ml of plasma, which included the percentage of reads within a koala that were the original endogenous KoRV-A sequence and the percentage attributed to the minor groups as predictors. Predicted values from linear regression model are shown with 95% confidence intervals. B) Summary of significant sole predictors of log10 KoRV *pol* copies per ml of plasma as determined by univariate linear regression modelling. Positive relationships are shown in green, while negative relationships are orange. The width of the link reflects the strength (R^2^) of the relationship: Age = 0.06 (Δ AIC = NA, p = 0.036); Number of subtypes = 0.06 (Δ AIC = 21.85, p = 0.003); Non-functional load proportion = 0.07 (Δ AIC = 19.57, p<0.001); Minor groups load proportion = 0.07 (Δ AIC = 21.74, p = 0.003); KoRV-D load proportion = 0.02 (Δ AIC = 26.27, p = 0.039), Minor groups + (Δ AIC = 24.54, p = 0.015); KoRV-B + (Δ AIC = 24.62, p = 0.015); Original KoRV-A load proportion = 0.18 (Δ AIC = 0, p<0.001); KoRV-A load proportion = 0.17 (Δ AIC = 2.29, p < 0.001).

In the best model predicting KoRV load from the KoRV subtype measures (S2 Table; as ranked by Akaike information criterion (AIC), where all predictors were significant, Δ AIC of second-best model = 2.5), KoRV load decreased as the proportion of original KoRV-A reads increased in the koalas (p < 0.001; S5 Fig) and KoRV load increased as the proportion of minor groups increased (p = 0.036; Fig 2A). The proportion of reads that were the original KoRV-A sequence was also the best sole predictor of KoRV load. The R^2^ value for the model incorporating both the proportion of original A and proportion of minor groups was 0.21. There were several other KoRV subtype measures that were significantly associated with KoRV load; however, their predictive strength was significantly weaker compared to proportion original KoRV-A (Figs 2B and S6).

### The association between KoRV parameters and secondary disease pathologies

**Fig 3:**
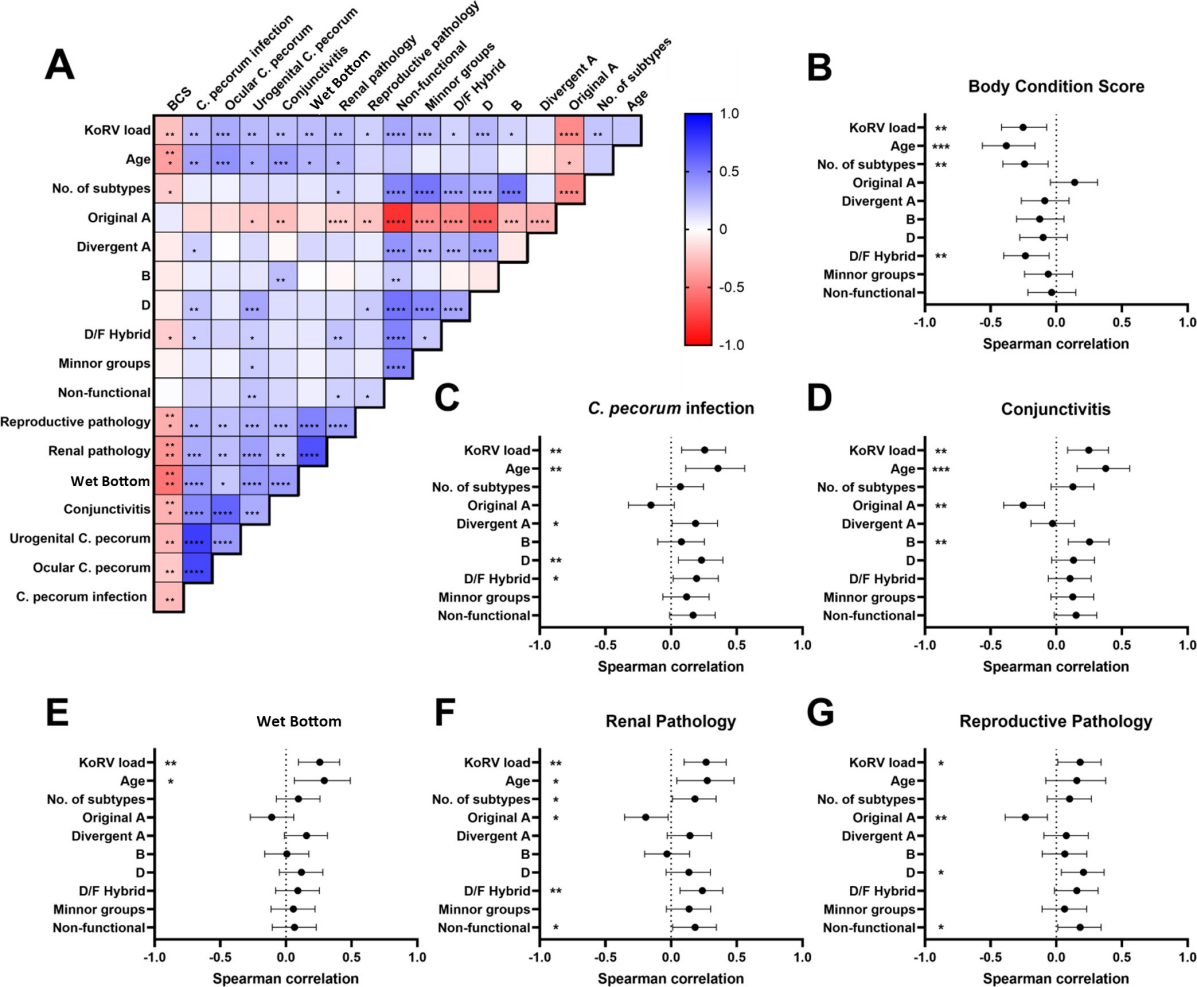
(A) Spearman correlation matrix, with colours representative of strength of correlation; Blue = positive correlation, Red = negative correlation. (B-G) Individual disease pathologies and Spearman correlation data for KoRV parameters; bars represent 95% confidence interval for Spearman correlation. *p* values * = <0.05, ** = <0.01, *** = <0.001, **** = <0.0001.

In a preliminary analysis that demonstrates the high level of interdependence in the dataset and how the KoRV and disease parameters covaried, individual associations were assessed by spearman correlation (Fig 3). KoRV load was significantly associated with each and every disease condition, *C*. *pecorum* infection and poor body condition score (BCS). Older age also appeared to be associated with disease, *C*. *pecorum* infection and poor BCS (Fig 3). It was also clear from the analysis that koalas suffering from one disease pathology were significantly more likely to have other pathologies, *C*. *pecorum* infection and poor BCS (Fig 3). In addition to KoRV load, other KoRV parameters, including the number of subtypes, proportion of reads that were the non-KoRV-A subtypes, proportion of reads divergent KoRV-A and proportion of non-functional reads were all associated with different disease pathologies (Fig 3).

As all the KoRV parameters were highly correlated, generalised logistic regression models, ranked by Akaike information criterion, were used to establish the best single KoRV predictors and combination of predictors for each of the disease parameters. Age was not included as a predictor in the formal model ranking analysis as it was only available for approximately half the sampled koalas resulting in less statitistical power. Instead, for each disease pathology, age and the best KoRV predictor identified by the model ranking were included as predicotrs in a post-hoc analysis to establish whether or not the observed disease-KoRV associations could be attributed to an underlying affect of age.

#### Body condition score

**Fig 4:**
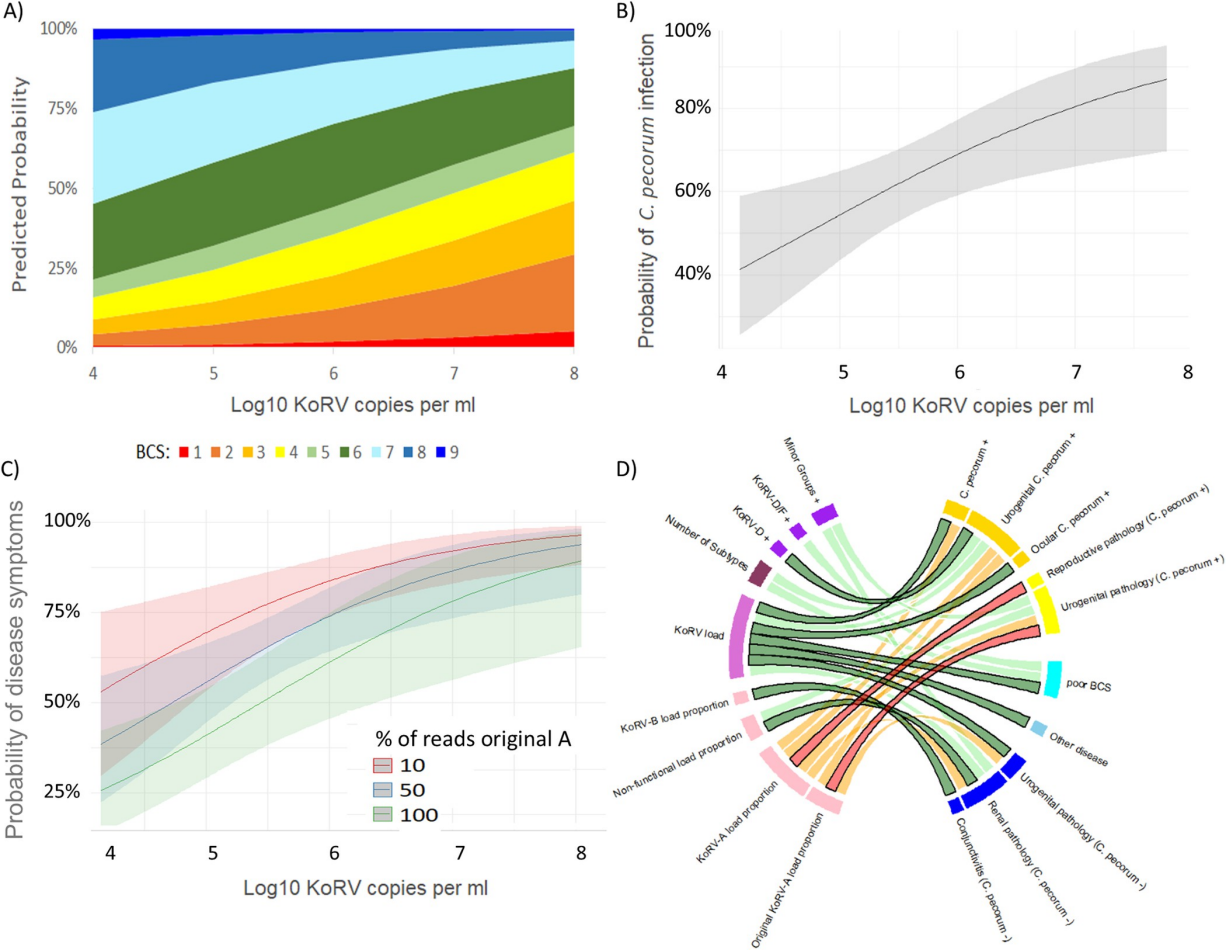
(A) The probability of a koala having each body condition score (BCS) as predicted by log10 KoRV pol copies per ml of plasma, which was a significant predictor in the two best models of BCS. Predicted values were produced from the best ordinal regression model averaged across KoRV-D/F positive and negative koalas. (B)The best model of the probability that a koala was infected with C. pecorum, in which log10 KoRV pol copies per ml of plasma was the sole predictor. Predicted values were produced from the binomial regression model, with the 95% confidence interval shown. (C) The best model of the probability of a koala presenting with disease symptoms, in which log10 KoRV pol copies per ml of plasma and the proportion of reads within a koala that clustered with the original KoRV-A sequence were predictors. Predicted values were produced from the best binomial regression model, with 95% confidence intervals shown. (D) Summary of significant sole KoRV predictors (left) of secondary disease measures (right) as determined by binomial regression modelling. The best sole predictor for each measure is indicated by the black outlined links, while links for all other significant predictors are not outlined. Green links show positive associations, while negative associations are shown in red and orange.

The koala body condition score (BCS) used by CWH is a measure of a koala’s overall condition based on the mass of muscle tissue around the scapula that ranges from 1 to 10, with scores below 5 being very poor [39]. Among the koalas sampled in this study, BCSs of 1 to 9 were observed (mean = 4.95; SD = 1.98). There were two closely ranked ordinal regression models that best explained the variation in BCS (Δ AIC = 0.31). In the first of these models (Nagelkerke’s R^2^ = 0.14), KoRV-load was found to be the best predictor for BCS, with a koala more likely to have a low BCS if their KoRV load was high (Fig 4A; p = 0.001). Additionally, koalas that were positive for KoRV-D/F were more likely to have low BCS than KoRV-D/F negative koalas (p = 0.008; S7 Fig). In the second model (Nagelkerke’s R^2^ = 0.13), KoRV load again predicted BCS and koalas were more likely to have high BCSs if they had fewer subtypes (p = 0.008) or a higher proportion of non-functional sequences (p = 0.023). There were no other significant predictors of BCS.

In post-hoc multiple ordinal regression, both koala age and KoRV load were significant predictors of BCS (p < 0.001 and p = 0.014, respectively), indicating that the association between BCS and KoRV load was not due to an underlying effect of age. The Nagelkerke’s R^2^ value for the model incorporating both age and KoRV load was 0.31.

#### C. pecorum infection

The best binomial regression model of whether a koala had *C*. *pecorum* (including either urogenital or ocular *C*. *pecorum*), included KoRV load and proportion KoRV-A as predictors (p = 0.006 and p = 0.05, respectively). Koalas with higher KoRV loads were more likely to be positive for *C*. *pecorum*, with the predicted probability of *C*. *pecorum* infection increasing decidedly from 40% to over 80% over the observed range of KoRV load (odds ratio increase per log change in KoRV load = 1.86; Figs 4B and S8). The probability of *C*. *pecorum* infection also increased as the proportion of KoRV-A reads decreased (Δ AIC = 4.28; p = 0.050), while none of the other KoRV predictors were significant. The probability of *C*. *pecorum* infection also significantly increased with age (tooth wear class; odds ratio increase per class = 1.44, p = 0.006). When age was included as a predictor in the best AIC ranked model, KoRV load was not a significant predictor of *C*. *pecorum* infection (p = 0.219). However, this lack of significance can primarily be attributed to the small number of samples (n = 66) for which both *C*. *pecorum* status and age were known, as KoRV load remained non-significant for the data subset when age was removed from the model (p = 0.119).

When binomial regression modelling was conducted specifically for urogenital *C*. *pecorum*, the best model, koalas were more likely to test positive for urogenital *C*. *pecorum* if they had higher KoRV loads (p = 0.01) or were positive for KoRV-D (p = 0.03). Further, whether or not a koala tested positive to KoRV-D (p = 0.02) was the best sole predictor of urogenital *C*. *pecorum* infection, followed closely by KoRV load (Δ AIC = 0.90; p = 0.008). Koalas were also significantly more likely to test positive for urogenital *C*. *pecorum* as the proportion of original KoRV-A (Δ AIC = 1.28; p = 0.009) or total KoRV-A (Δ AIC = 1.59; p = 0.01) reads decreased or as the number of subtypes increased (Δ AIC = 4.30; p = 0.046; Fig 4D).

The probability of a koala testing positive for ocular *C*. *pecorum* infection increased as KoRV load increased (p < 0.001; Fig 4D). There were no other significant predictors of ocular *C*. *pecorum* infection.

In post-hoc multiple logistic regression of *C*. *pecorum* infection as predicted by age and KoRV load, only KoRV load was found to be a significant predictor (p = 0.0015). The pseudo R^2^ value (Tjur’s R^2^) for the model was 0.03, while the R^2^ value for collinearity between age and KoRV load was 0.036.

#### Overall disease pathology

The best two binomial regression models of whether a koala exhibited disease symptoms of any kind (S1 Table, ID:12; where all predictors were significant) showed that the probability of disease symptoms increased as KoRV load increased (p = 0.001) and displayed an additive increase as the proportion of either original KoRV-A decreased (1^st^ model, p = 0.005; Figs 4C and S9) or the proportion of total KoRV-A decreased (2^nd^ model, Δ AIC = 0.61). In the first model, the predicted probability of a koala having disease symptoms (when the proportion of KoRV-A reads was 90%) increased strikingly from 25% to over 85% within the range of observed KoRV load (odds ratio increase per log change in KoRV load = 2.92; Fig 4C). In agreement with this, the best sole predictor of disease symptoms was KoRV load, followed by original KoRV-A proportion (Δ AIC = 3.60). A koala was also significantly more likely to show disease symptoms as total KoRV-A proportion decreased (Δ AIC = 5.08; p < 0.001), as the number of subtypes (Δ AIC = 14.60; p < 0.002), KoRV-D read proportion (Δ AIC = 17.66; p < 0.024) or non-functional read proportion (Δ AIC = 18.36; p < 0.018) increased and when the koalas were positive for the minor groups (Δ AIC = 15.78; p < 0.004).

In post-hoc multiple logistic regression of disease as predicted by age and KoRV load, only KoRV load was found to be significant (p = 0.0015 for KoRV load and p = 0.10 for age). The pseudo R^2^ value (Tjur’s R^2^) for the model was 0.23, while the R^2^ value for collinearity between the two predictors was 0.054, indicating that the effect of KoRV load is relatively independent of age.

#### Individual disease pathologies

Of those koalas that were positive for urogenital *C*. *pecorum*, the proportion of original KoRV-A reads was the best predictor of whether or not they exhibited urogenital pathology (i.e. wet bottom, reproductive pathology and/or renal pathology; p = 0.004; S1 Table, ID = 7), with those koalas that had higher proportions less likely to exhibit pathology. *C*. *pecorum* positive koalas were also significantly less likely to show urogenital pathology as their total KoRV-A read proportion increased (Δ AIC = 0.86; p < 0.007), as their non-functional sequence proportion (Δ AIC = 3.76; p = 0.041) or the number of subtypes decreased (Δ AIC = 2.94; p = 0.021), or if they were negative for the KoRV minor groups (Δ AIC = 4.17; p = 0.043; Fig 4D). When considering the different urogenital pathologies separately for *C*. *pecorum* positive koalas, there were no significant predictors of wet bottom or renal pathology, while the only significant predictor of reproductive pathology was the total KoRV-A read proportion (p = 0.032; Fig 4D).

Of those koalas that were negative for urogenital *C*. *pecorum*, KoRV load was the best predictor of whether or not they exhibited urogenital pathology (p = 0.013), with those koalas that had higher loads more likely to show disease symptoms. *C*. *pecorum* negative koalas were also significantly more likely to show urogenital pathology as the proportion of original KoRV-A decreased (Δ AIC = 2.24; p = 0.034). When considering the different urogenital pathologies separately for *C*. *pecorum* negative koalas, there were no significant predictors of wet bottom or reproductive pathology. However, whether or not a koala showed evidence of renal pathology was significantly explained by KoRV load (p = 0.023), non-functional read proportions (p = 0.013), total KoRV-A read proportions (p = 0.047) and whether or not they had any of the KoRV minor groups (p = 0.043; Fig 4D).

For koalas that were positive for ocular *C*. *pecorum*, there were no significant KoRV predictors of whether or not they had conjunctivitis. However, koalas that tested negative for ocular *C*. *pecorum*, were marginally more likely to have conjunctivitis if they had higher read proportions for KoRV-B (p = 0.049).

The probability of a koala presenting with disease pathologies other than those associated with Chlamydia (S1 Table, ID = 11) was also found to be associated with increased KoRV load (p = 0.045; Fig 4D) but no other KoRV measures were found to be significant predictors.

The presentation of koalas with de-pigmentation of the skin on their front and rear paws has been anecdotally considered to be a KoRV pathology. However, we did not find a significant association between paw de-pigmentation and any of our KoRV predictor variables.

## Discussion

Our large-scale study of wild koalas in rehabilitation has confirmed previous suggestions that there is an association between KoRV and secondary disease [6–8]. The koalas that presented at Currumbin Wildlife Hospital suffered from a large number of distinct disease pathologies, many of which co-occurred and were often symptoms of *C*. *pecorum* infection. Assessing how each of these pathologies were associated with KoRV necessitated numerous statistical tests and as such we focus on the consistent patterns across analyses, while the findings of individual tests require further confirmation. In contrast to the small scale study of Waugh et al. [7], we reveal that KoRV load as opposed to the presence of any particular subtype is most strongly associated with a range of disease measures. KoRV *pol* copies per ml of plasma was the best predictor of more disease measures than any other KoRV variable including for the general measures of koala health (body condition score and overall probability of disease). Koala age, as assessed by tooth wear class, was also strongly associated with disease and KoRV load did significantly increase with age. Nonetheless, KoRV load remained a significant predictor of body condition, *C*. *pecorum* infection and disease symptoms after accounting for the effect of age. Further, the associations of KoRV load with disease had large effect sizes, with a one log increase in KoRV load increasing the odds of a koala presenting with disease pathologies to the same extent as nearly 3 (2.9) years of age. This highlights the major role KoRV may play in the high prevalence of disease in northern koala populations.

Despite these strong associations between secondary disease and KoRV load, a causative association cannot be ascribed. As this was a purely observational study, we are unable to exclude the possibility that high KoRV load is a consequence of disease or that the observed associations are the result of underlying common factors. Genetic and/or environmental factors could potentially impact immune function and thereby increase KoRV load as well as the likelihood of *C*. *pecorum* infection and disease pathologies. To resolve the nature of the relationship between KoRV and secondary disease, longitudinal observational studies and/or interventional trials using antiretrovirals to supress KoRV activity would be required. Such studies are challenging and resources intensive, however, the strong associations identified here suggest that such studies would be worthwhile.

Despite the important caveat regarding causation, our findings are consistent with general knowledge from other retroviruses that would suggest that higher circulating KoRV load within blood serum is indicative of higher levels of immune cell infection and immune suppression. Sarker et al. [40] showed that KoRV infection of HEK293T cells led to activation of pathways associated with viral infection, such as cytokine receptor interactions and interferon signalling pathways. In other retroviruses, including HIV, infection promotes dysregulation of autophagy in lymphocytes limiting their ability to produce cytokines and respond to pathogens [41]. One study that artificially infected human peripheral blood mononuclear cells (PBMCs) with KoRV, suggested dysregulation of effector cytokines [42]. Such exacerbation of cytokine dysregulation and impairment of immune function with increasing KoRV load is a likely biological mechanism underlying the observed associations with secondary diseases.

Our results show that KoRV load increases as the relative abundance of the exogenous subtypes increase. We found a negative correlation between KoRV load and the proportion of *env* deep sequencing reads that were the original endogenous KoRV-A sequence. This is consistent with the partial suppression of endogenous KoRV-A expression, while the exogenous KoRV subtypes obtain high infection/replication levels. It has recently been shown that a piRNA response is triggered against endogenous KoRV-A within germline cells [21]. In *C*. *elegans*, piRNAs establish silent epigenetic states that are maintained for many generations. KoRV-A silencing in the germline may therefore be propagated into adult T-cells, suppressing retroviral expression. Nonetheless, a 4-log variation in the original KoRV-A sequence expression was observed between koalas (calculated as KoRV load multiplied by the proportion of sequencing reads) and relative abundance of exogenous subtypes only accounted for a small proportion of the variation in KoRV load. Therefore, given the strong association between KoRV load and secondary disease, determining the underlying factors that lead to higher levels of KoRV expression and viremia will undoubtedly aid in future koala rehabilitation efforts.

KoRV-B has previously been associated with secondary disease [6,7], while until now there has been little information on the disease association of the other exogenous subtypes. In this study, KoRV-B, KoRV-D, KoRV-D/F, exogenous KoRV-A, minor groups and even non-functional sequences were all found to be positively associated with one or more disease measures. Thus, we have revealed strong correlations between all the exogenous subtypes and secondary disease as well as with high levels of viremia. These correlations point toward a potentially indirect effect of the exogenous subtypes whereby they replicate to higher viremia and that higher viremia is associated with secondary disease. However, our use of AIC model ranking, allowed us to disentangle the direct effect of the non-KoRV-A subtypes from that of KoRV load. In our best models, once the effect of KoRV load was accounted for, the original KoRV-A sequence remained a significant negative predictor of overall disease probability and koalas that were positive for KoRV-D or KoRV-D/F were more likely to have urogenital *C*. *pecorum* infection or low BCS, respectively. Thus, our findings suggest that the exogenous subtypes may indeed increase susceptibility to and exacerbate secondary diseases through other mechanisms besides higher levels of activity/viremia. This is in contrast to the small scale study of Quigley *et al*. [28] that found that KoRV-D expression was higher in healthy compared to diseased koalas. This discrepancy many be the result of the sample size of that study or among population differences, as KoRV-D is known to be highly diverse [24]. A longitudinal study by Robbins *et al*. [29] also did not find an association between KoRV subtype prevalence, read proportions or diversity and which koalas became infected with *C*. *pecorum* or progressed to exhibiting disease pathologies. However, that study was based on proviral profiles rather than circulating vRNA and it may be that vRNA reveals associations not evident from DNA (which is dominated by the endogenous KoRV-A sequences [24,29,30]).

Our findings do not support the conclusions of previous studies that KoRV-B in particular is associated with *Chlamydial* disease. A small scale study by Waugh et al. (3) and a longitudinal study by Chaban et al. [6] found that koalas that were KoRV-B positive were more likely to have *Chlamydial* disease. Though, Chaban et al. [6] found no association between any measure of KoRV and *C*. *pecorum* infection. By contrast, we have shown that KoRV-B is only associated with conjunctivitis in the absence of *C*. *pecorum* infection and was not a significant predictor of any other *Chlamydia* associated pathology or *C*. *pecorum* infection. Yet we found several other significant predictors of *Chlamydia* associated pathology and *C*. *pecorum* infection. These differences may potentially be attributable to the resolution of the different studies. Firstly, both previous studies determined KoRV-B positivity by PCR. This method is less sensitive than deep sequencing and does not detect all KoRV-B variants [24,30], such that only a subset of KoRV-B positive koalas with comparatively high KoRV-B proviral loads would have been detected in those studies. Additionally, neither previous study assessed the presence or abundance of the other non-KoRV-A subtypes. This could have led to the conclusion that KoRV-B was associated with *Chlamydial* disease when in fact their findings could reflect higher non-KoRV-A proviral loads.

The findings from this study provide recommendations for the conservation, management and treatment of koalas in regard to KoRV infection. Firstly, based on previous reports of KoRV-B virulence, several zoos within Australia have attempted to limit the transmission of KoRV-B within their captive populations by quarantining KoRV-B PCR positive koalas (per. coms). However, our finding that all exogenous KoRV sequences and subtypes are associated with secondary disease, suggests that preventing the transmission of individual subtypes by quarantining infected individuals is unlikely to be successful. Whereas, reducing the overall prevalence of exogenous KoRV may be possible through targeted breading, as we have and others have previously shown that the transmission of the exogenous subtypes is predominately from mother to offspring [23,32]. Additionally, as most subtypes are geographically restricted [19], limiting interbreeding between populations may help to reduce subtype diversity. Secondly, our finding that KoRV *pol* copies per ml of plasma is most strongly associated with secondary disease pathologies suggests that research, rehabilitation and conservation programs should target understanding what drives inter individual differences in KoRV load. If the factors that increase KoRV replication can be identified, then strategies to combat these factors can be developed to reduce KoRV load and within host KoRV diversity, which is likely generated through ongoing recombination and mutation during viral reintegration and transcription. We argue that such an approach is more likely to successfully improve individual and population health than attempting to eliminate particular subtypes.

## Materials and methods

### Ethics statement

Sample collection was carried out under approval from the University of Queensland’s Native/Exotic Wildlife and Marine Animals Ethics Committee (AE36153).

### Sample collection

Blood samples were collected from 151 koalas during admittance to Currumbin Wildlife Hospital, Australia, for treatment and rehabilitation between January 2018 and June 2019. Up to 3 mls of blood was drawn by a qualified veterinarian from the cephalic vein of each koala during conscious restraint. The blood was then transferred to an EDTA coated tube to prevent clotting. The blood was then centrifuged at 16 000 g for 10 minutes and the plasma collected by pipetting and stored at -80°C.

### Assessment of disease pathologies

The sampled koalas originated from wild populations in south-east Queensland and were admitted due to trauma or disease. Detailed veterinary records were collected on each koala’s condition at the time of admittance and during treatment. These records were used to score the koalas for the presence and extent of a set of gross disease pathologies as outlined in S1 Table. The koala’s body condition score (BCS) was determined from the mass of muscle tissue around the scapula and ranged from 1 (very poor) to 10 (excellent) [39]. A koala was identified as having ‘wet bottom’ by the presence of discoloured fur on their rump with or without the presence of fresh urine. This is an external indication of urinary leakage and cystitis. Renal pathology was identified by the presence of abnormalities in the bladder, kidneys or ureters on ultrasound, such as thickening of the bladder wall. Reproductive pathology was identified by the presence of abnormalities in the testes, prostrate, uterus or ovaries on ultrasound. Urogenital pathology was identified as the presence of wet bottom, renal and/or reproductive pathology. Conjunctivitis was visually identified by the presence of inflammation of the transparent membrane of the eyelid and eyeball of either or both eyes. Neoplasia was putatively diagnosed by abnormal cytology indicative of lymphoma or the presence of tumours. Confirmation of neoplasia was not completed due to the small number of potential cases in the dataset. Oxalate Nephrosis was identified by the presence of oxalate crystals in the urine in addition to renal pathology. A small number of other disease pathologies were also identified such as respiratory infections and infected wounds. If a koala was diagnosed with any disease pathology listed, then they were classified as showing ‘disease pathology’.

The majority of koalas were also tested by Currumbin Wildlife Hospital for the presence of urogenital and ocular *Chlamydia pecorum* infection using loop-mediated isothermal amplification [37]. *C*. *pecorum* infection can cause urogenital pathologies and conjunctivitis. However, not all instances of these disease pathologies were diagnosed in *C*. *pecorum* positive koalas in this study. Therefore, when investigating the association of KoRV with wet bottom, renal pathology, reproductive pathology and urogenital pathology we assessed *C*. *pecorum* positive and negative koalas separately to reflect the likely differences in the underlining cause of the symptoms.

### RNA Extraction and cDNA synthesis

Total RNA was extracted from the plasma samples using the High Pure Viral Nucleic Acid Kit (roche) according to the manufacturer’s instructions with modifications as outlined in Blyton et. al. [19] First strand cDNA was created using Superscript III (Invitrogen) as per Blyton et. al. [19].

### Plasma viral loads

Plasma viral loads were estimated by quantitative PCR of a 110 bp fragment of the KoRV *pol* gene amplified in triplicate [36]. Each 10 μl reaction contained 1X SYBR Green PCR Master Mix, each primer at 100 nM and 1 μl of a 10-fold dilution of the template cDNA. Amplification, standard curves and quality control was carried as per Blyton et. al. [19] with melt curves visually inspected to ensure target specificity. All replicates and samples had a strong primary peak at ~81°C, while 11% (52/459) reactions had a small secondary peak at ~75°C (S10 Fig). The number of KoRV *pol* molecules present in each reaction and ml of plasma were calculated from the average CT values and standard curves using the appropriate standard equations.

### KoRV subtype profiles

An approximately 500 bp region of the KoRV *ENV* gene containing the previously identified hypervariable domain as described in Chappell et al. [24] was amplified and sequenced on the Illumina Nextseq. The resulting data was then processed in CLC genomics workbench 20 and QIIME 2 [43] according to Blyton et. al. [19] to determine which subtypes were carried by each koala and in what proportions. The paired reads were merged according to standard parameters (Mismatch cost = 2, Minimum score = 8, Gap cost = 3, Maximum unaligned end mismatches = 5) and unmerged reads discarded. The merged reads that were not of sufficient quality (limit = 0.05) or contained greater than two ambiguous bases were then removed. Finally, only sequences that were between 450 and 600 bp were retained. The processed sequences were rarefied to 10 000 reads per sample, with the exception of two samples that had few than this number of reads. The rarefied reads were then *de novo* clustered across all samples at 97% similarity in QIIME 2 [43]. Clusters containing only a single read across the dataset were discarded. The representative sequences from the remaining clusters were then blasted using default parameters against the NCBI ‘nt’ database in CLC genomics workbench 20. Sequences that were not identified as koala retrovirus (based on the accession of the match with the lowest E-value) were discarded from further analysis.

To determine if the representative KoRV sequences were putatively functional, they were translated *in silico* using CLC genomic workbench 20. The correct frame was determined by identifying the presence of conserved motifs in the receptor binding domain of the translated sequences. In general a +3 frame produced protein sequences that aligned with those of the known subtypes [24]. Sequences containing missense mutations or deletions of greater than 4 amino acids were then identified and designated as non-functional. To identify the presence of frameshift mutations, the similarity of the representative amino acid sequences before and after the hypervariable domain were compared to the original A sequence [38]. If the difference in amino acid percentage similarity between the beginning and end of the sequence was greater than 25% the representative sequence was considered to have a frameshift mutation and be non-functional.

Subtype designations for the intact representative KoRV sequences were assigned by a three-step process. Firstly, the nucleotide sequences of the hypervariable domain and flanking motifs (corresponding to aa81-143 of KoRV A numbering) of the representative sequences were aligned in CLC to those of references sequences for each of the previously identified subtypes (KoRV-A [44]; KoRV-C (AB828005) [25]; KoRV-E [26]; KoRV-B21, D35, F9, G4, H2, I4 [24]; KoRV-K [23]. The representative sequences were considered to belong to the subtype of the reference sequence to which they showed the highest similarity if that similarity was greater than 80% and the second most similar reference sequence was more than 10% less similar than the first. Secondly, the genetic nucleotide distances among the reference and representative sequences were calculated in CLC. Principle components analysis (PCoA) was then performed using the resulting distance matrix with the vegan package [45] in R [46]. Distinct sequence groups were identified from the first three axes of variation and assigned subtype designations from the presence of a reference sequence in that group. In general, the subtype designations from method one and two were in good agreement. Thirdly, the translated sequences of the hypervariable domain of each PCoA group were aligned with the reference sequences in CLC and visually examined to confirm their subtype designation. PCoA groups that were not assigned to a previously identified subtype were also confirmed as novel subtypes or hybrid sequences by examination of the aligned amino acid sequences. The read proportions of the subtypes was then inferred from the representative sequence by sample table generated from QIIME 2 clustering.

### Association between plasma viral loads and subtype diversity

To determine how total KoRV load is related to the different KoRV subtypes and their relative abundances, linear regression models were performed using the stats package in R [46]. Log10 transformed plasma viral load was the response variable in the analysis and the other KoRV variables listed in S2 Table were predictor variables together with koala sex and age. Separate models were constructed for all combinations of one to three predictor variables and select combinations of four variables. The proportions of reads that were each of the subtypes were not included as explanatory variables in the same model as the presence/absence of each of the subtypes. The different models were then ranked by Akaike information criterion to establish the best single predictors and combination of predictors. Models that contained non-significant predictors were disregarded. Shapiro–Wilk tests were also performed to confirm that the model residuals conformed to the assumption of normality.

### The association between KoRV parameters and secondary disease

To establish if and how KoRV loads and the different subtypes are associated with secondary disease, regression models were constructed in R with each of the disease measures listed in S1 Table as response variables. Ordered logistic regression was used to assess body condition score using the MASS package [47], while, generalised linear models with logit link functions were used to assess the other variables. For each response variable the KoRV parameters listed in S2 Table were used as predictors with separate additive models constructed for all combinations of one to three predictor variables and select combinations of four variables (interactions between variables were not considered; S3 Table). The proportions of reads that were each of the subtypes were not included as explanatory variables in the same model as the presence/absence of each of the subtypes. The different models were then ranked by Akaike information criterion to establish the best single predictors and combination of predictors. Models that contained non-significant predictors were disregarded.

## Supporting information

S1 Fig In silico translated amino acid sequences of the env hypervariable region of A/D (a), A/F (b), B/D (c) and ten examples of D/F intermediate sequences aligned against relevant reference sequences.
(TIF)

S2 Fig In silico translated amino acid sequences for the env hypervariable region of Group 1 (a), Group 2 (b), Group 3 (c) and Group 4 (d) aligned against the reference sequences for the previously identified subtypes.
(TIF)

S3 Fig Percentage of KoRV-A reads within koalas that clustered with the original endogenous KoRV-A sequence at 97% nucleotide identity. (TIF)

S4 Fig log10 KoRV *pol* copies per ml of plasma by koala tooth wear class Black points indicate data points. Blue line indicates fitted regression relationship.
(TIF)

S5 Fig log10 KoRV *pol* copies per ml of plasma by the percentage of reads within a koala that clustered with the original KoRV-A sequence. (TIF)

S6 Fig log10 KoRV *pol* copies per ml of plasma by the number of KoRV subtypes/groups (excluding KoRV-A and the non-functional sequences). Open points indicate data points. Blue line indicates fitted regression relationship.
(TIF)

S7 Fig (a) Body condition score in relation to log10 KoRV *pol* copies per ml of plasma for KoRV-D/F positive and negative koalas. (b) The probability of a koala having each body condition score as predicted by log10 KoRV *pol* copies per ml of plasma and the proportion of reads within a koala that were KoRV-D/F. Predicted values were produced from the best ordinal regression model, with 95% confidence intervals shown.
(TIF)

S8 Fig a) Boxplot of log10 KoRV *pol* copies per ml of plasma by whether or not the koalas presented with disease symptoms. b) Boxplot of the percentage of reads within a koala that clustered with the original KoRV-A sequence by whether or not the koalas presented with disease symptoms.
(TIF)

S9 Fig Boxplot of log10 KoRV *pol* copies per ml of plasma by whether or not the koalas had *Chlamydia pecorum*. (TIF)

S10 Fig Melt curves for KoRV *pol* qPCRs of cDNA generated from total RNA extracted from cell free plasma of 153 koalas. All reactions had a strong peak at ~81 C. 52 reactions in run 1 had a small secondary peak at ~75C, while the remaining 230 reactions in run 1 (b) and all 177 samples in run 2 (c) had a very small or no secondary peak.
(TIF)

S1 Table Demographic and disease measures recorded for sampled koalas. (PDF)

S2 Table KoRV variables measured and tested for their association with secondary disease. (PDF)

S3 Table List of KoRV parameter models for predicting disease pathologies. (XLSX)

S1 Data Supplementary data file containing the KoRV parameters and disease pathology scores for each koala included in the study. (XLSX)
