# Supplementary figures and images for: Koala retrovirus load and non-A subtypes are associated with secondary disease among wild northern koalas
Source: PLoS Pathog. 2022 May 19;18(5):e1010513. doi: 10.1371/journal.ppat.1010513 (PMC9119473; doi:10.1371/journal.ppat.1010513)

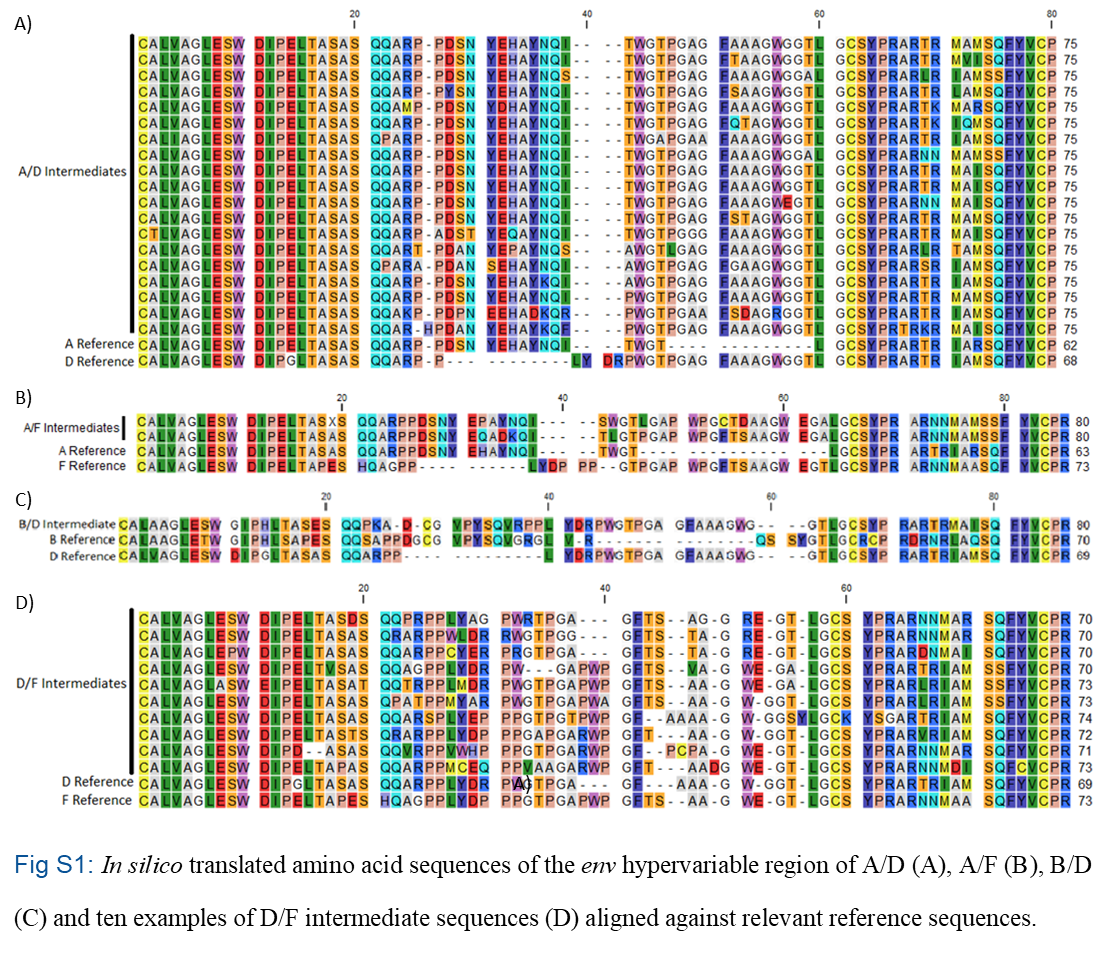

Supplement: S1 Fig — In silico translated amino acid sequences of the env hypervariable region of A/D (a), A/F (b), B/D (c) and ten examples of D/F intermediate sequences aligned against relevant reference sequences. (TIF) [file ppat.1010513.s001.tif]

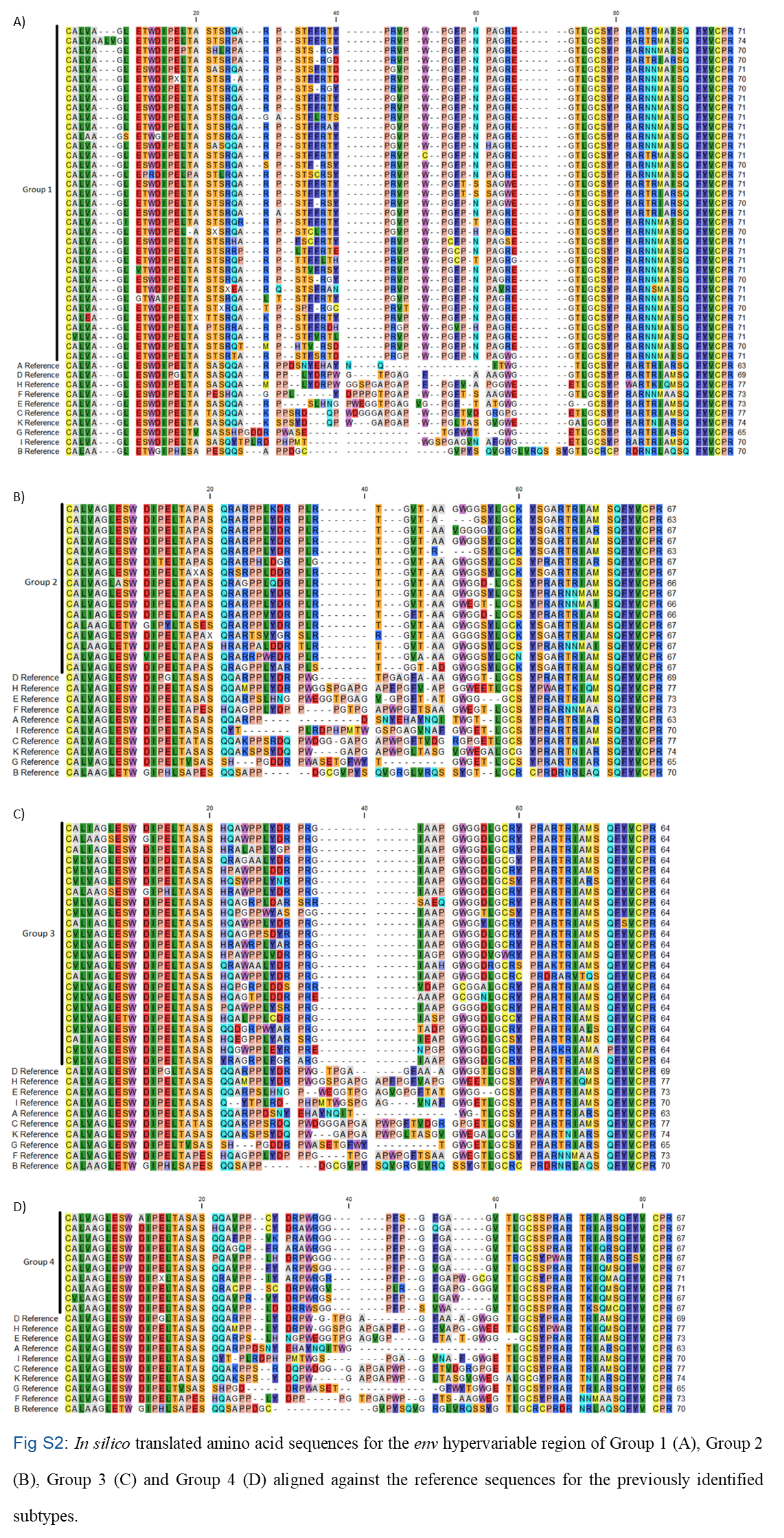

Supplement: S2 Fig — In silico translated amino acid sequences for the env hypervariable region of Group 1 (a), Group 2 (b), Group 3 (c) and Group 4 (d) aligned against the reference sequences for the previously identified subtypes. (TIF) [file ppat.1010513.s002.tif]

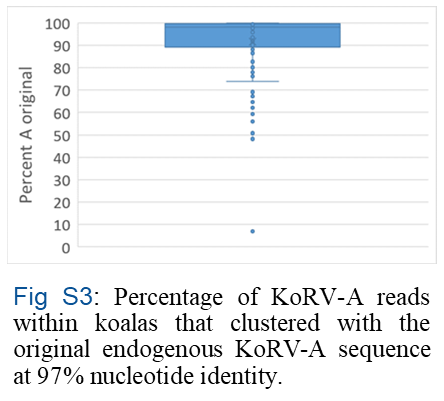

Supplement: S3 Fig — (TIF) [file ppat.1010513.s003.tif]

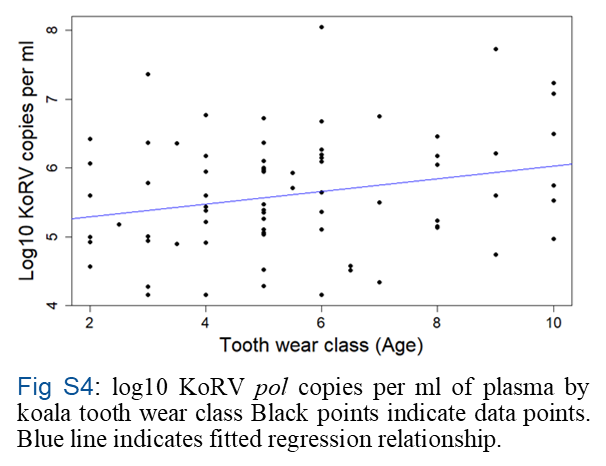

Supplement: S4 Fig — Blue line indicates fitted regression relationship. (TIF) [file ppat.1010513.s004.tif]

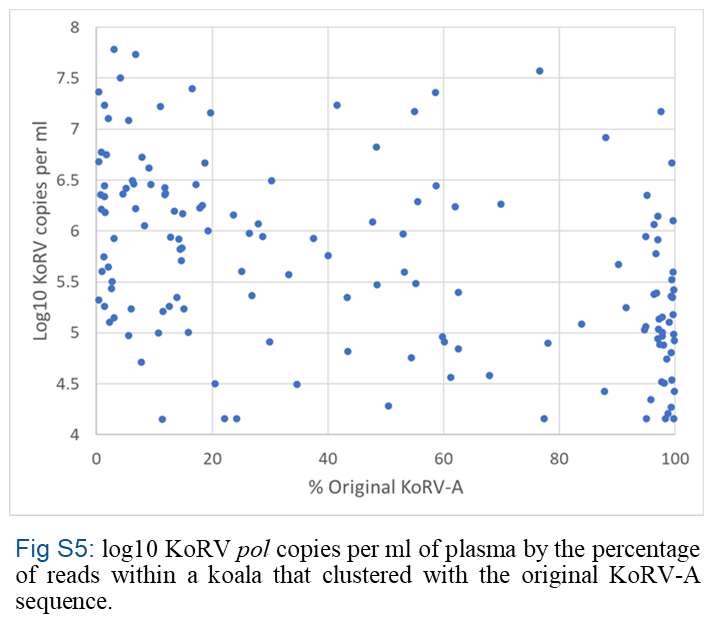

Supplement: S5 Fig — (TIF) [file ppat.1010513.s005.tif]

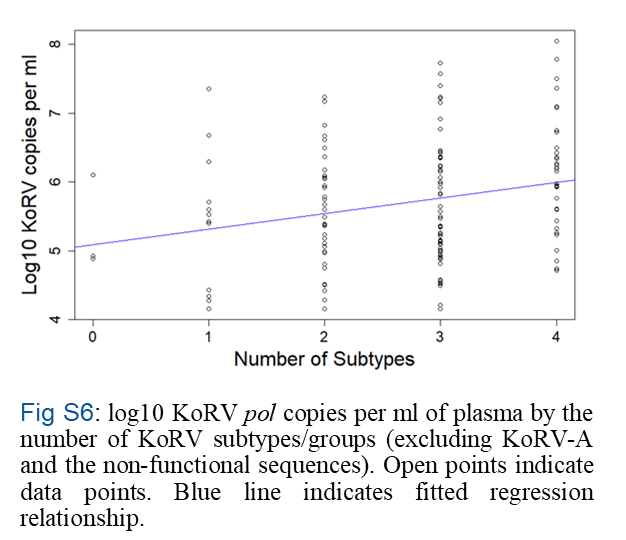

Supplement: S6 Fig — Open points indicate data points. Blue line indicates fitted regression relationship. (TIF) [file ppat.1010513.s006.tif]

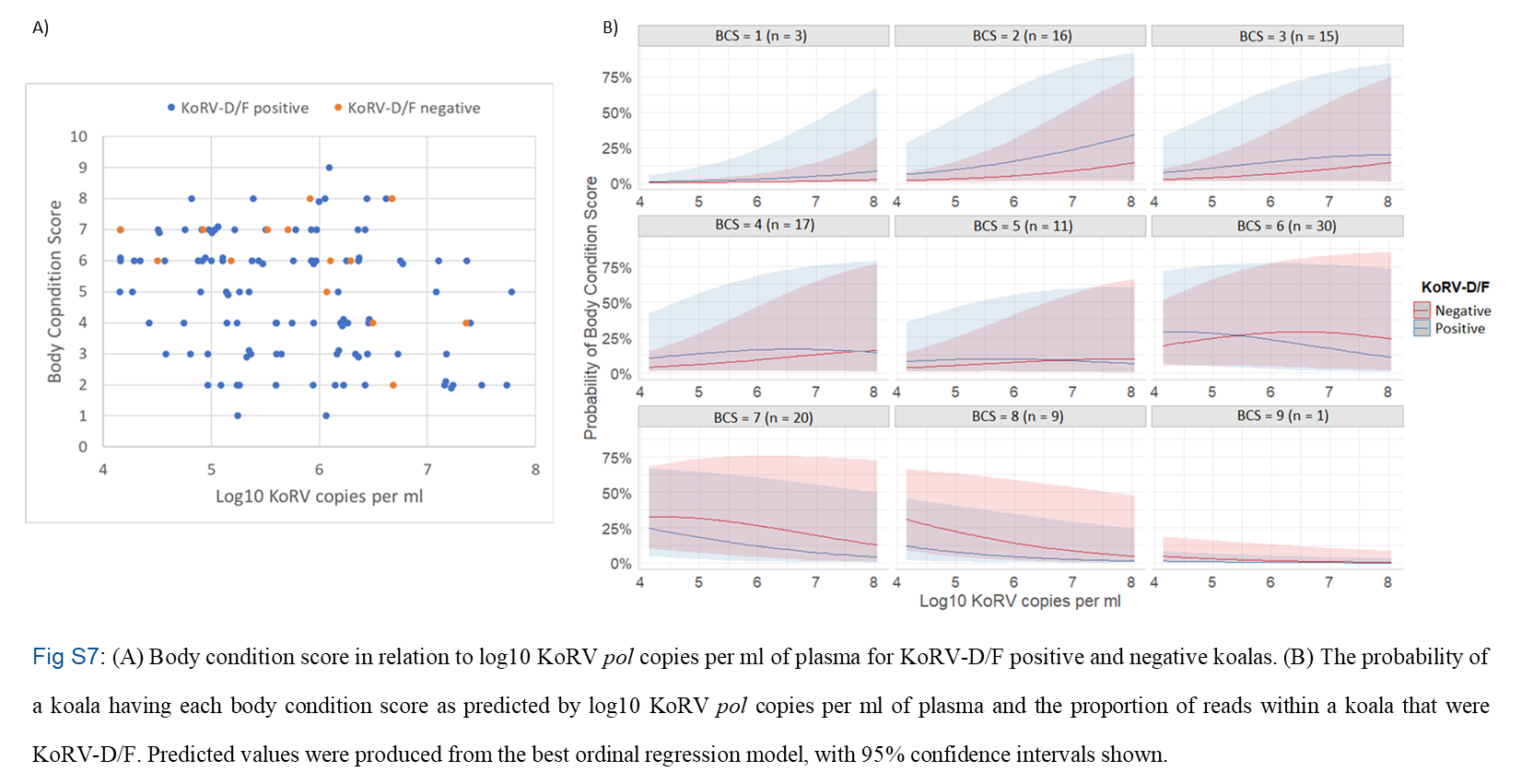

Supplement: S7 Fig — (a) Body condition score in relation to log10 KoRV pol copies per ml of plasma for KoRV-D/F positive and negative koalas. (b) The probability of a koala having each body condition score as predicted by log10 KoRV pol copies per ml of plasma and the proportion of reads within a koala that were KoRV-D/F. Predicted values were produced from the best ordinal regression model, with 95% confidence intervals shown. (TIF) [file ppat.1010513.s007.tif]

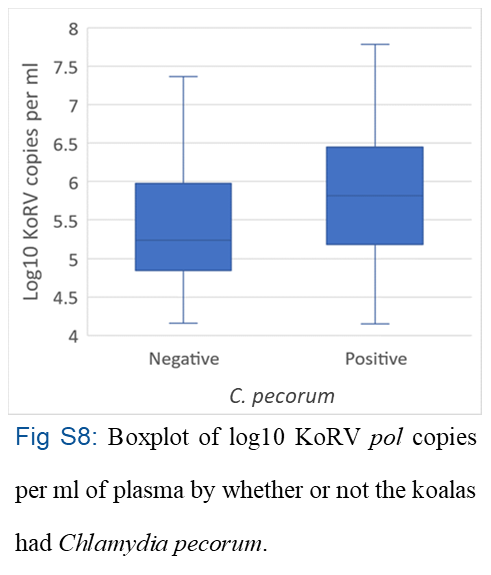

Supplement: S8 Fig — a) Boxplot of log10 KoRV pol copies per ml of plasma by whether or not the koalas presented with disease symptoms. b) Boxplot of the percentage of reads within a koala that clustered with the original KoRV-A sequence by whether or not the koalas presented with disease symptoms. (TIF) [file ppat.1010513.s008.tif]

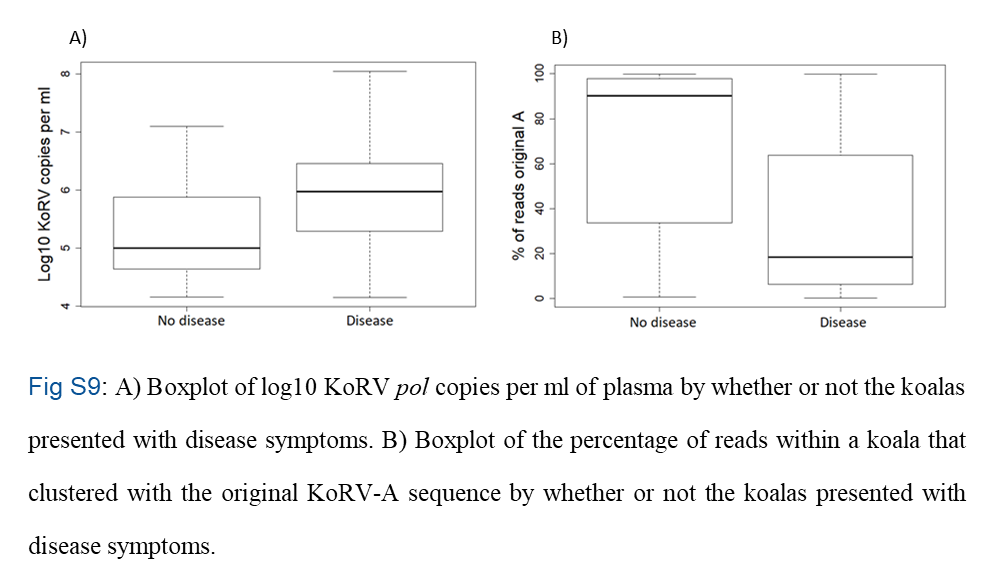

Supplement: S9 Fig — (TIF) [file ppat.1010513.s009.tif]

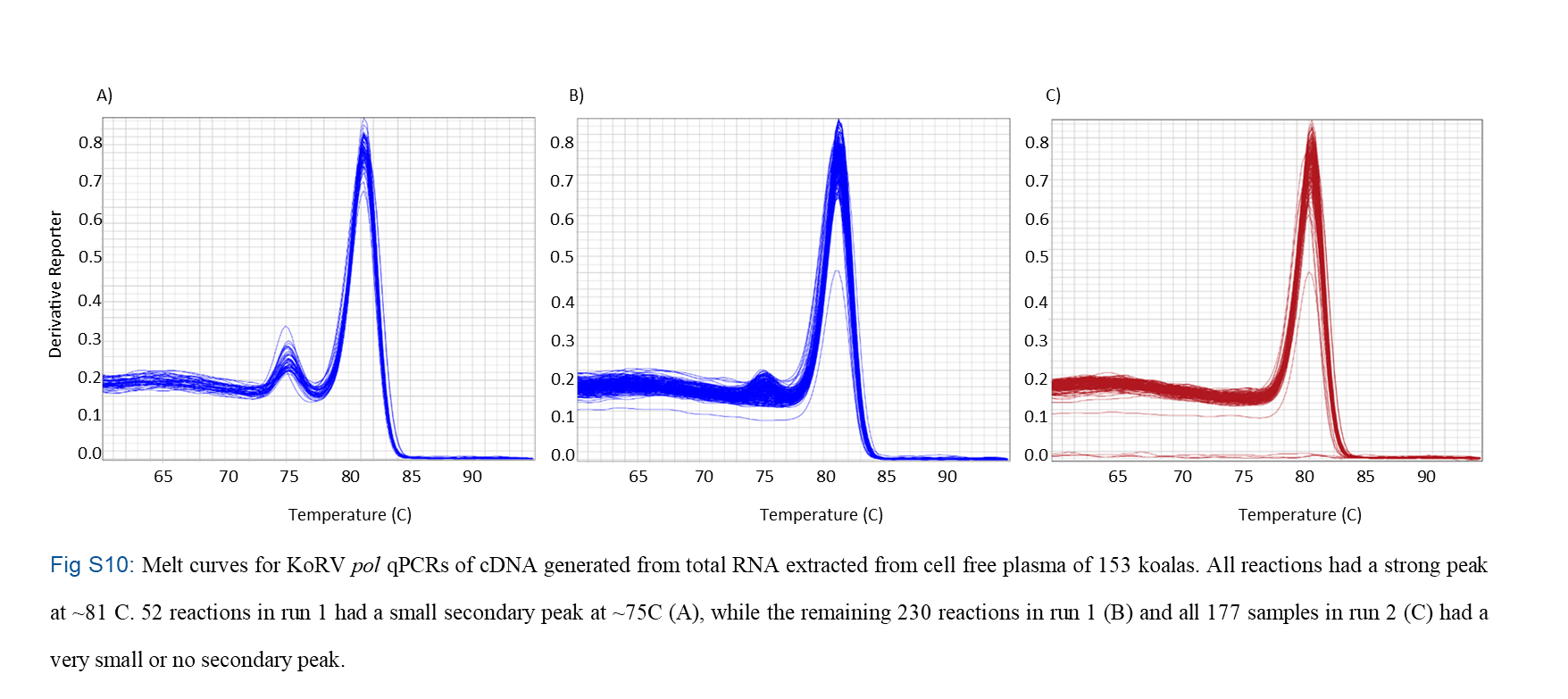

Supplement: S10 Fig — All reactions had a strong peak at ~81 C. 52 reactions in run 1 had a small secondary peak at ~75C, while the remaining 230 reactions in run 1 (b) and all 177 samples in run 2 (c) had a very small or no secondary peak. (TIF) [file ppat.1010513.s010.tif]
