# Supplementary material for: Koala retrovirus load and non-A subtypes are associated with secondary disease among wild northern koalas
Source: PLoS Pathog. 2022 May 19;18(5):e1010513. doi: 10.1371/journal.ppat.1010513 (PMC9119473; doi:10.1371/journal.ppat.1010513)
Supplement: S1 Table — (PDF) [file ppat.1010513.s011.pdf]

Table S1: Demographic and disease measures recorded for sampled koalas

ID	Variable	Available Records	Positive Cases (%)	Type of measure
**1**	Sex	149	N/A	Binary
**2**	Tooth wear (Age)	81	N/A	Integer
**3**	Body Condition Score (BCS)	124	N/A	Ordinal
**4**	Wet bottom	145	49 (33.8)	Binary
**5**	Renal pathology[1]	138	61 (44.2)	Binary
**6**	Reproductive pathology[1]	141	36 (25.5)	Binary
**7**	Urogenital pathology (4,5 &6)[2]	143	78 (54.5)	Binary
**8**	Conjunctivitis	149	56 (37.6)	Binary
**9**	Neoplasia[3]	151	5 (3.3)	Binary
**10**	Oxalate nephrosis[3]	151	5 (3.3)	Binary
**11**	Other Disease (including 9)[4]	151	26 (17.2)	Binary
**12**	Disease pathology (7, 8, 10 & 11)[2]	151	100 (66.2)	Binary
**13**	Urogenital *C. pecorum* infection	125	59 (47.2)	Binary
**14**	Ocular *C. pecorum* infection	128	60 (46.8)	Binary
**15**	*C. pecorum* infection (13 & 14)[2]	130	81 (62.3)	Binary
**16**	De-pigmented paws	151	11 (7.3)	Binary

1. Determined by an experienced veterinarian from ultrasound examination of anatomical structures
2. Compound variable produced from combining variables listed in brackets
3. The association between this disease measure and KoRV measures was not statistically tested as there were too few positive cases for reliable assessment.
4. The classification of ‘other disease’ refers to a heterogeneous assortment of conditions including (but not limited to) respiratory infections, infected wounds and neoplasia.
