# Supplementary material for: Koala retrovirus load and non-A subtypes are associated with secondary disease among wild northern koalas
Source: PLoS Pathog. 2022 May 19;18(5):e1010513. doi: 10.1371/journal.ppat.1010513 (PMC9119473; doi:10.1371/journal.ppat.1010513)
Supplement: S2 Table — (PDF) [file ppat.1010513.s012.pdf]

Table S2: KoRV variables measured and tested for their association with secondary disease

Variable		Type
Log10 KoRV *pol* copies per ml of plasma		Continuous
Number of Subtypes[1]		Integer
Proportion of reads:	Original A	Continuous
	A	Continuous
	B	Continuous
	D	Continuous
	D/F Intermediates	Continuous
	Minor Groups[2]	Continuous
	Non-functional	Continuous
Presence/Absence of:	B	Binary
	D	Binary
	D/F Intermediates	Binary
	Minor Groups[2]	Binary

1. KoRV-A and Non-functional sequences were not included in this count as they were present in all samples. 'Minor Groups' were considered a single 'subtype' for the purposes of this count.
2. Minor groups = subtype H, the four new sequence groups and the undefined sequences
